# Screening of sustainable supply chain performance evaluation indicators based on the ill-conditioned index cycle method

**DOI:** 10.1371/journal.pone.0293038

**Published:** 2024-03-04

**Authors:** Qili Deng, Xing Huang, Jiang Zou, Yicheng He

**Affiliations:** 1 School of Modern Business, Mianyang City College, Mianyang, Sichuan, China; 2 School of Economics and Management, Southwest University of Science and Technology, Mianyang, Sichuan, China; 3 Institute of Western China Economic Research, Southwest University of Finance and Economics, Chengdu, Sichuan, China; King Khalid University, SAUDI ARABIA

## Abstract

The establishment of an evaluation indicator system that can accurately assess the sustainability of a supply chain while further enhancing its performance is vital and relevant. Based on the connotation of sustainable supply chains and triple bottom line theory, indicators are initially proposed from economic, environmental, and social dimensions. To increase the explanatory power of the indicator system and decrease information redundancy, the coefficient of variation is applied to identify the indicators with weak interpretation intensity, the ill-conditioned index cycle method is utilized to filter out indicators with redundant information, and data on 100 Chinese listed companies from 2019 to 2021 are used as samples. A performance evaluation indicator system of sustainable supply chains with 16 indicators is ultimately established. The information interpretation strength index and cumulative information contribution rate verify the rationality of the final indicator system. The outcome demonstrates that this screening method can strengthen the representativeness of the indicator system and rapidly reduce redundancy, leading to the better discrimination of the evaluation results. The findings of this study provide an indicator system and a methodological reference for both companies and policymakers and can aid in the transformation of supply chains toward sustainability.

## Introduction

The economy and society are currently experiencing a period of accelerated formation of a new "dual circulation" development pattern. The implementation of sustainable supply chains with low emissions, high responsibility, and increased transparency has become a critical basis for ensuring the stability and security of enterprise development. The establishment of a scientific and practical performance evaluation system for sustainable supply chains has emerged as a crucial challenge that numerous enterprises must overcome. Recently, research on performance indicator systems of sustainable supply chains has been an active field among academics. However, while studies on the influencing factors have been conducted, practice reveals that these aspects cannot be clearly distinguished from the assessment results, and it remains difficult to improve the representativeness and explanatory power of indicator systems. Therefore, this study aims to further conduct in-depth research on the screening of indicators of sustainable supply chain performance.

Research related to the establishment of sustainable supply chain performance indicator systems has focused on the factors that influence performance and the methods for screening indicators. Present research on the influencing factors has mainly concentrated on the aspects of conventional supply chain performance evaluation models or triple bottom line (TBL) theory. For example, Haghighi et al. [1] proposed 12 indicators by combining a balanced scorecard with key performance indicators from four factors: financial, customer, internal operations, and learning and growth. Huang and Wen [2] merged the performance prism model with the supply chain operations reference model to select 54 performance indicators for sustainable catering industry supply chains from economic, social, ecological, and resource factors. Based on TBL theory, Vivas et al. [3] presented a set of 19 indicators, Cao and Xiong [4] developed a set of 31 indicators, Dai et al. [5] proposed 16 indicators, while Aunyawong et al. [6] found that the sustainable supply chain performance evaluation should be focused on economic performance, social performance, environmental performance as well as institutional performance.

A few studies have also provided indicator systems based on scenarios for sustainable implementation. For example, Zhao and Chen [7] developed 28 indicators based on lean, agile, flexible, and green strategies, and constructed the LARG-P index. Wu and Zhu [8] proposed 10 indicators with which to evaluate the sustainability of supply chains for platform-based logistics companies from economic, willingness, social, flexible, and ecological factors. Qorri et al. [9] offered 15 indicators from environmental, social, supply chain operation, and economic factors to reveal the correlation between sustainable supply chain practices and enterprise performance. Chen et al. [10] established a performance benchmark for sustainable supply chain with 16 indicators based on the revenue-cost-sharing contract. Attia [11] developed 20 indicators for the assessment of the performance of Egyptian textile enterprises based on sustainable supply chain management, customer relationship management, and competitive advantages.

Current research on indicator screening methods primarily addresses the significance and information redundancy of indicators. Common methods for the selection of important indicators include entropy, rough sets, grey correlation analysis, the coefficient of variation (*CV*), and Bayesian network algorithms. For example, Erol et al. [12] eliminated indicators with limited information contribution using fuzzy information entropy and chose 37 pertinent indicators for the performance evaluation of sustainable supply chains. Narimissa et al. [13] selected 88 key indicators to construct a sustainable supply chain evaluation system based on the Delphi method and meta-synthesis approach. Tseng et al. [14] built a sustainable supply chain performance evaluation system by weighting 57 indicators using the fuzzy Delphi method and the analytic hierarchy process (AHP). Li et al. [15] established an indicator system of green shipbuilding supply chain applying the specific application of improved analytic hierarchy process and fuzzy comprehensive evaluation. He et al. [16] prioritized the importance of five key variables for the assessment of the performance of supply chains using TOPSIS and grey management. Hsu et al. [17] identified 25 indicators and chose the top five that affected the performance of sustainable supply chains with the fuzzy Delphi approach.

Methods of screening for redundant indicators include the Pearson correlation coefficient, backpropagation (BP) neural networks, factor analysis, R-type cluster analysis, and matrix reflecting image correlation. For example, Dong et al. [18] eliminated indicators with higher correlation in the primary selection using the Pearson correlation coefficient. Kuang et al. [19] utilized partial correlation analysis and analysis of variance to locate redundant indicators and selected 48 indicators with which to assess the financial supply chains of small and medium-sized companies. Zheng and Li [20] integrated rough set reduction with a BP network to minimize duplicate information and built a dynamic supply chain performance evaluation system. Wang and Deng [21] used factor analysis to select the four most representative indicators out of 26 to evaluate the performance of the agricultural supply chain. Cao and Fan [22] applied principal component (PCA) analysis to identify the three most representative indicators from eight input indicators, and the two most relevant indicators from six output indicators, to evaluate the performance of the green supply chain of agricultural products. Rodriguez [23] combined comprehensive PCA with multiple correspondence analysis to screen out nine indicators and established a green supply chain performance evaluation system.

Based on the literature review, the existing findings are relatively systematic in terms of the influencing factors and screening methods, providing a basis and methodological inspiration for this study. However, there remains a lack of rationality in the screened indicator systems, making it challenging to differentiate between assessment results. This is due to two reasons. First, due to their disregard for the implementation details of environmental and social factors, the indicator systems lack comprehensiveness and representativeness. The value of the evaluation results is thus not immediately apparent. Second, current approaches for key indicator screening are overly reliant on subjective judgments, and they fail to effectively reduce the redundancy of the indicator set. Present methodologies stress the correlation between every two indicators as the basis for information redundancy screening. It appears random to exclude one of the two indicators with a strong correlation, so it is easy to retain the indicator with low information interpretation intensity, which lessens the relevance of the findings of the assessment and compromises the objectivity of the evaluation. Given this, in the present study, indicators are extracted based on the connotation of sustainable supply chains and the TBL theory from the three dimensions of the economy, the environment, and society. The *CV* is applied to identify key indicators with strong interpretation intensity, and the ill-conditioned index cycle method is utilized to rapidly reduce the overall information redundancy. The issues of the weak explanatory power and low representativeness of the indicators are addressed, as are the problems of differentiating between the evaluation results, thus providing an evaluation basis and method reference for enterprises and policymakers.

## Indicator selection

### Mass selection

The concept of sustainable supply chains integrates sustainable development theory into supply chain management and aims at the coordination of economic, environmental, and social benefits in various business processes to achieve the sustainable development of enterprises [24]. TBL theory requires companies to balance their pursuit of economic interests with their obligations to the environment and society [25]. The logical framework for assessing supply chain sustainability is thus built on economic, environmental, and social factors [26]. Economic sustainability is attained by streamlining management costs and elevating output effectiveness in manufacturing, warehousing, distribution, and other sectors [27]. Environmental sustainability is achieved by limiting environmental consequences during the processes of material selection, production, and site recycling [28], and companies should inspect on environmental management within their firms [29]. Social sustainability is maintained by coordinating upstream and downstream organizations to comply with rules and regulations, and to fulfill obligations to stakeholders, such as customers, employees, and shareholders, to implement varied management and achieve a good societal reputation [30]. The interplay and mutual coordination of the TBL contribute to supply chain sustainability. Only by thoroughly assessing each of these three facets can the performance of the sustainable supply chain be evaluated in its entirety and with objectivity [31]. Therefore, in this study, sustainable supply chain performance indicators are extracted from the economic, environmental, and social dimensions based on the connotation of sustainable supply chains and TBL theory.

When selecting performance indicators, reference was first made to the Sustainability Reporting Guidelines issued by the United Nations Environment Program and the Global Reporting Initiative, which involve 79 indicators; the Social Responsibility Guidance Standard formulated by the International Organization for Standardization, ISO 26000, which involves 217 indicators; and the Environmental, Social, and Governance Reporting Guide published by the Hong Kong Stock Exchange, which involves 32 indicators. The indicators that could be identified in 392 articles from the past five years were then retrieved from the Web of Science and CNKI core collection. Then, the indicators cited by authoritative sources and those found in the literature were organized into three dimensions, and any indicators that were manifestly irrelevant to the subject were removed. Finally, indicators with similar meanings were merged, and 53 indicators were ultimately obtained.

### Initial selection

First, 21 indicators were removed by the principles of scientificity and representativeness that should be adopted when selecting indicators. These included seven economic indicators, namely the net profit growth rate, the sales net profit margin, the net operating asset net profit margin, the asset impairment rate, the negative tax rate, the current ratio, and the economic order. Moreover, nine environmental indicators were removed, namely environmental certification, the environmental protection tax burden rate, the environmental investment rate, the environmental product production rate, the environmental product profit margin, the high-energy-consuming product production rate, the high-energy-consuming product income ratio, supplier risk management, and the green supply chain management system. Finally, five social indicators were removed, including the high-quality development level, the business ethics level, internal satisfaction management, the employment ratio of fresh graduates, and the average employee training time. Second, 12 indicators were eliminated based on the principles of comparability and obtainability when selecting indicators, and data mining was conducted according to the financial statements and social responsibility reports from 100 core node companies of large-scale supply chains from 2019 to 2021. These indicators included five economic indicators, namely the growth rate of shareholder dividends, the product return rate, the new product development cycle, the production process site rate, and the stockout loss rate, and two environmental indicators, namely negative environmental events and the water resource consumption per unit of income. Five social indicators were also eliminated, namely the product qualification rate, product safety and quality, negative reputation events, poverty alleviation and regional development investment, and labor security system construction. Finally, 20 preliminary indicators were ultimately retained, as shown in Table 1.

**Table 1:** The preliminary indicators of sustainable supply chain performance evaluation.

Dimensions	Indicators	Type	Descriptions
**Economic**	Asset-Liability rate (*F1*)	N	Total liability / Total asset (Quantitative)
	Revenue growth rate (*F2*)	P	Revenue growth / Revenue of last period (Quantitative)
	Return on equity rate (*F3*)	P	Net profit / Average net asset (Quantitative)
	Capital accumulation rate (*F4*)	P	Equity growth / Equity of the beginning balance (Quantitative)
	Sustainable growth rate (*F5*)	P	Retained earnings growth / Retained earnings of the beginning balance (Quantitative)
	Inventory management (*F6*)	P	Operating costs / Average inventory (Quantitative)
	Logistics management (*F7*)	P	The rationality of product warehousing and transportation management. (Qualitative)
**Environmental**	Environmental management (*E1*)	P	Environmental supervision and assessment management. (Qualitative)
	Carbon intensity (*E2*)	N	Tons of carbon dioxide equivalent / Revenue (million RMB) (Quantitative)
	Energy consumption (*E3*)	N	Energy consumption of electricity, gas, and oil, etc. (Qualitative)
	Discharge and reuse (*E4*)	P	Management and execution of discharge and reuse during production and operation processes. (Qualitative)
	Product environmental impact (*E5*)	P	The impact of the products or services produced on the environment. (Qualitative)
	Renewable resource utilization (*E6*)	P	The utilization of renewable materials and energy in the production and operation process. (Qualitative)
**Social**	Compliance and business Ethics (*S1*)	P	Compliance with laws and regulations and anti-corruption measures. (Qualitative)
	Diversified collaborative governance (*S2*)	P	Diversified management models and collaborative governance of information technology. (Qualitative)
	Emergency and risk management (*S3*)	P	Emergency and risk management regulations and practice. (Qualitative)
	Health and safety of employee (*S4*)	P	The physical and mental health of employees and security guarantee. (Qualitative)
	Growth investment of employees (*S5*)	P	Investment in improving employee’s knowledge and skills. (Qualitative)
	Public welfare investment(*S6*)	P	Investment in poverty alleviation and public welfare, etc. (Qualitative)
	Social Responsibility rankings (*S7*)	P	Scoring of social responsibility reports by the China Securities Investment Fund Industry Association (Quantitative)

## Screening methodology

### Total concept

A preliminary indicator system was designed after some indicators were discarded based on the selection principles. There may still have been certain indicators with overlapping or insufficient explanatory power. The aim of this study to select the key indicators that can strike a balance between importance and effectiveness. The ill-conditioned index cycle method evolved from the concept of the condition index in econometrics, which accurately reflects the multicollinearity between variables. The higher the value, the more redundant information the variable contains. This method provides scientific support for effectively reducing the overall information redundancy of the indicator system. Compared with conventional correlation analysis techniques, it can efficiently decrease the information overlap while avoiding the random deletion of any indicator, which would result in excessive information deletion, affect the thoroughness of the evaluation data, and skew the evaluation results [32].

Following the principle of first considering the significance and then concentrating on correlation [33], if a redundant indicator is removed first, it is likely that too many weakly influencing indicators will be retained, which could result in the removal of crucial indicators and alter how assessment results are differentiated. To ascertain the value of indicators, weakly contributing indicators should thus first be identified and eliminated using the *CV*. The most redundant indicator is then iteratively deleted using the ill-conditioned index cycle method until the overall ill-conditioned index of the remaining indicator system is below the target threshold [32]. Finally, the cumulative information contribution rate (*CR*) and the information interpretation strength index (*IS*) are combined to verify the scientific validity and effectiveness of the methodology used in this study.

### Approach and steps

#### Step 1: Standardization of data

As the index system contains qualitative and quantitative indicators, positive and negative indicators, and indices with varied dimensions, the range transformation method is applied to standardize the raw data, as follows:

$$Positive\;indices:\;X_{ij}^{\prime }=\frac{X_{ij}-\min\left(X_{ij}\right)}{{max(X}_{ij})-min(X_{ij})}$$ (1)

$$Negative\;indices:\;X_{ij}^{\prime }=\frac{{max(X}_{ij})-X_{ij}}{{max(X}_{ij})-min(X_{ij})}$$ (2)

where *i* = 1, 2…, *n*, *j* = 1, 2…, *n*, *X*_*ij*_ is the original value of the *j*th sample of indicator *I*, and *X’*_*ij*_ is the standardized value of *X*_*ij*_. Moreover, *Max (X*_*ij*_*)* is the maximum value of indicator *I* among all samples, and *min (X*_*ij*_*)* is the minimum value of indicator *I*.

#### Step 2: Screening based on information contribution

The *CV* is a commonly used index in statistics to measure the degree of dispersion between data [34]. It can be used to screen indicators to determine how well each indicator can discriminate between different types of information. The larger the *CV*, the stronger the discriminative ability of the indicator, and vice versa. This screening round aims to streamline the indicator system by eliminating indicators that contribute little information to the evaluation outcomes. According to statistics, the data tend to be evenly distributed when the *CV* is less than 0.3. An indicator does not significantly contribute information to the evaluation results if the observed value of the indicator is uniformly distributed. Therefore, the *CV* information contribution screening threshold was set as 0.3 in this study [35]. *CV* is calculated as follows.


$${CV}_{i}=\frac{\sqrt{\frac{1}{n-1}\sum_{i=1}^{n}(X_{ij}^{\prime }-\frac{1}{n}\sum_{i=1}^{n}X_{ij}^{\prime }{)}^{2}}}{\frac{1}{n}\sum_{i=1}^{n}X_{ij}^{\prime }}$$ (3)

After applying standardized data to Eq (3), the indicators are screened according to how much information they contribute, and if their *CV* is less than 0.3, they should be removed. The process then moves on to the following step.

#### Step 3: Determination of the redundancy degree

After filtering the information contribution, the ill-conditioned index measures the redundancy level of the remaining indicator system. The standardized matrix A of values is used when calculating the ill-conditioned index of the indicator system (*CI*_*n*_). The eigenvalues λ_1_, λ_2_,…, λ_n_, of matrix *A*^*T*^*A* are calculated by the characteristic Eq (4), where *A*^*T*^ is the transpose matrix of the standardized matrix *A*, and *E*_*n*_ is the identity matrix.


$$|A^{T}A-\lambda_{j}E_{n}|=0$$ (4)

After obtaining the maximum eigenvalue *λ*_*max*_ and the minimum eigenvalue *λ*_*min*_, the ill-conditioned index *CI*_*n*_ is calculated according to Eq (5).


$${CI}_{n}=\sqrt{\lambda_{max}/\lambda_{min}}$$ (5)

According to econometrics theory, when *CI*_*n*_ *<* 10, there is no multicollinearity between variables; when 10 ≤ *CI*_*n*_ < 15, there is weak multicollinearity between variables; when 15 ≤ *CI*_*n*_ < 30, there is moderate collinearity between variables; when 30 ≤ *CI*_*n*_ < 100, there is strong multicollinearity between variables; when *CI*_*n*_ ≥ 100, there is severe multicollinearity between variables [36]. The issue of "sustainability" in the supply chain is closely interwoven; if there is no overlap between the indicators, the objective evaluation results might not be complete. The screening aims to lessen information overlap rather than entirely eradicate it [37]. The information contribution rate must simultaneously be considered while applying this screening approach. Therefore, the target threshold of the ill-conditioned index was set as 15 in this study [38]. After contribution screening in Step 2, if the ill-conditioned index of the remaining indicator set is higher than 15, the process moves on to redundancy screening in Step 4.

#### Step 4: Screening based on information redundancy

**Fig 1:**
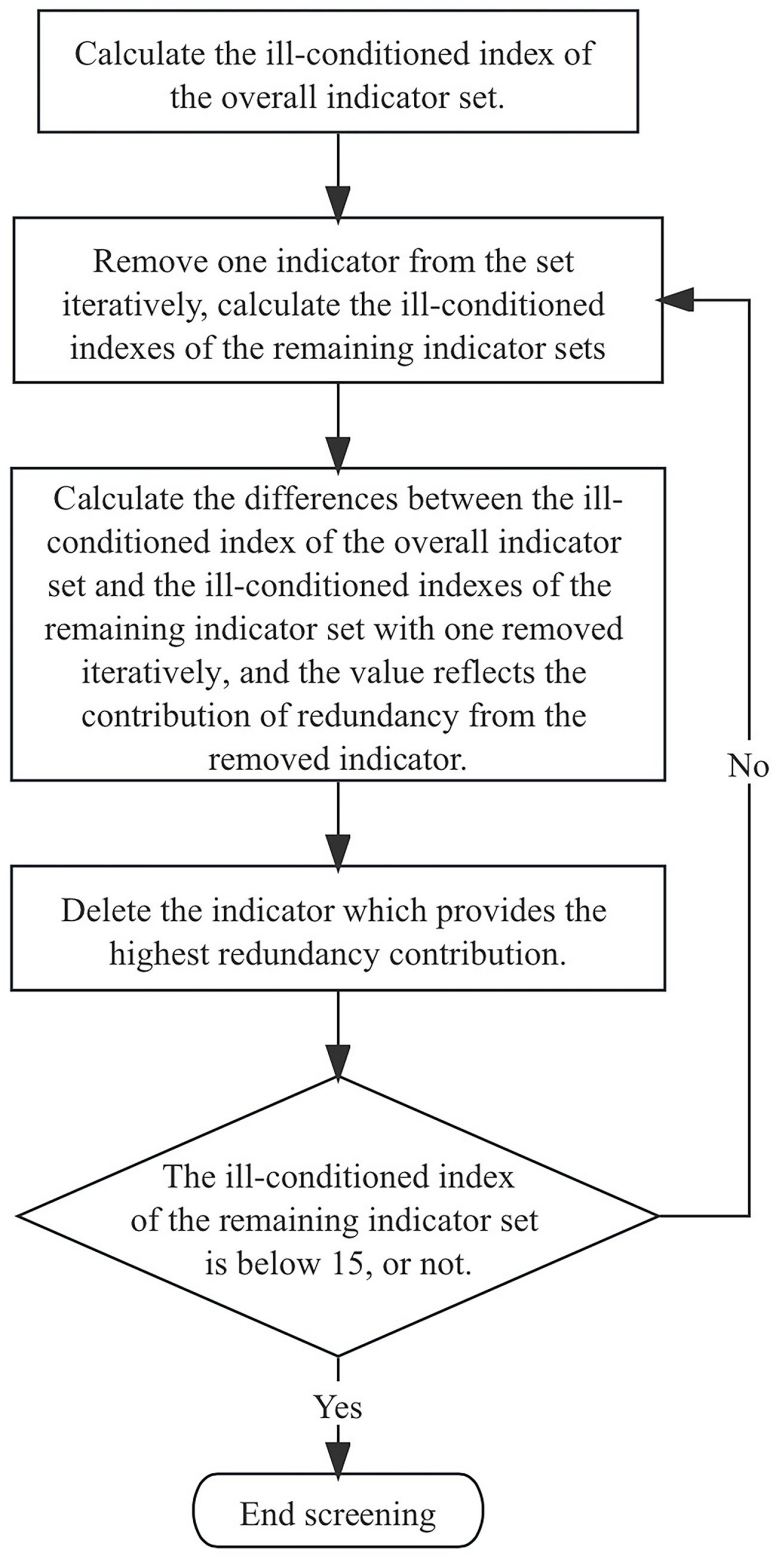
The process of redundancy screening.

Obtaining the degree of redundancy of the indicator system alone is not helpful for screening, as the specific contribution of each indicator to the whole evaluation system cannot be identified. When screening, first, one of the indicators is removed at a time, and the ill-conditioned indexes both with and without the removed indicators are calculated. Second, the difference between the two ill-conditioned indexes is calculated to reflect how each removed indicator adds redundancy to the evaluation system. Third, the indicator that contributes the most to redundancy is deleted. This process is repeated until the ill-conditioned index of the remaining indicator system is below the target threshold. The process is shown in **Fig 1.**

To quantify how each indicator (*F*_*i*_) contributes to redundancy, the ill-conditioned index (*CI*_*s*_) of the indicator set with *F*_*i*_ is calculated, as is the ill-conditioned index (*CI*_*s-1*_) of the indicator set with *F*_*i*_ alone removed. The contribution of indicator *F*_*i*_ to the information redundancy in its indicator set is represented by *CI*_*fi*_.

$$CI_{fi}=CI_{s}‐CI_{s‐1}$$ (6)

where *s = 1*, *2*…, *n*, *F*_*i*_ is any of the indicators, *CI*_*s*_ is the ill-conditioned index of *S* indicators that include *F*_*i*_, and *CI*_*s-1*_ is the ill-conditioned index of *S-1* indicators without *F*_*i*_ alone.

A higher *CI*_*fi*_ demonstrates that the indicator *F*_*i*_ has contributed more to the redundancy in the indicator system. Therefore, the indicator corresponding to the maximum *CI*_*fi*_ in the current indicator system should be deleted. The screening can end if the ill-conditioned index (*CI*_*s-1*_) of the remaining indicator system falls below the target threshold. Otherwise, this procedure is repeated until the value falls below the target threshold.

#### Step 5: Rationality test

To avoid the excessive deletion of indicators and compromising the thoroughness of the evaluation information, *CR* and *IS* are combined in this study to evaluate the rationality of the screening results. From the concept of PCA theory, if the principal components contribute more than 85%, most of the information in the original indicator system is reflected [39]. In this study, the information contribution is quantified based on *CV*, and *CR* reflects the information content of the screened indicator system as compared to the initial indicator system. If *CR* is more than 85%, it is considered that the filtered indicator system is reasonable. The equation of *CR* is as follows:

$$CR=\frac{\sum_{i=1}^{s}CV}{\sum_{i=1}^{k}CV}$$ (7)

where *k = 1*, *2*…, *n*.

To further illustrate the information interpretation intensity of the screened indicator system, *IS* is introduced, as given by Eq (8).


$$IS=\frac{\frac{1}{S}\sum_{i=1}^{S}\frac{\sum_{j=1}^{n}({X^{\prime }}_{ij}-\bar{X_{ij}}{)}^{2}}{n-1}}{\frac{1}{K}\sum_{i=1}^{K}\frac{\sum_{j=1}^{n}({X^{\prime }}_{ij}-\bar{X_{ij}}{)}^{2}}{n-1}}$$ (8)

**Fig 2:**
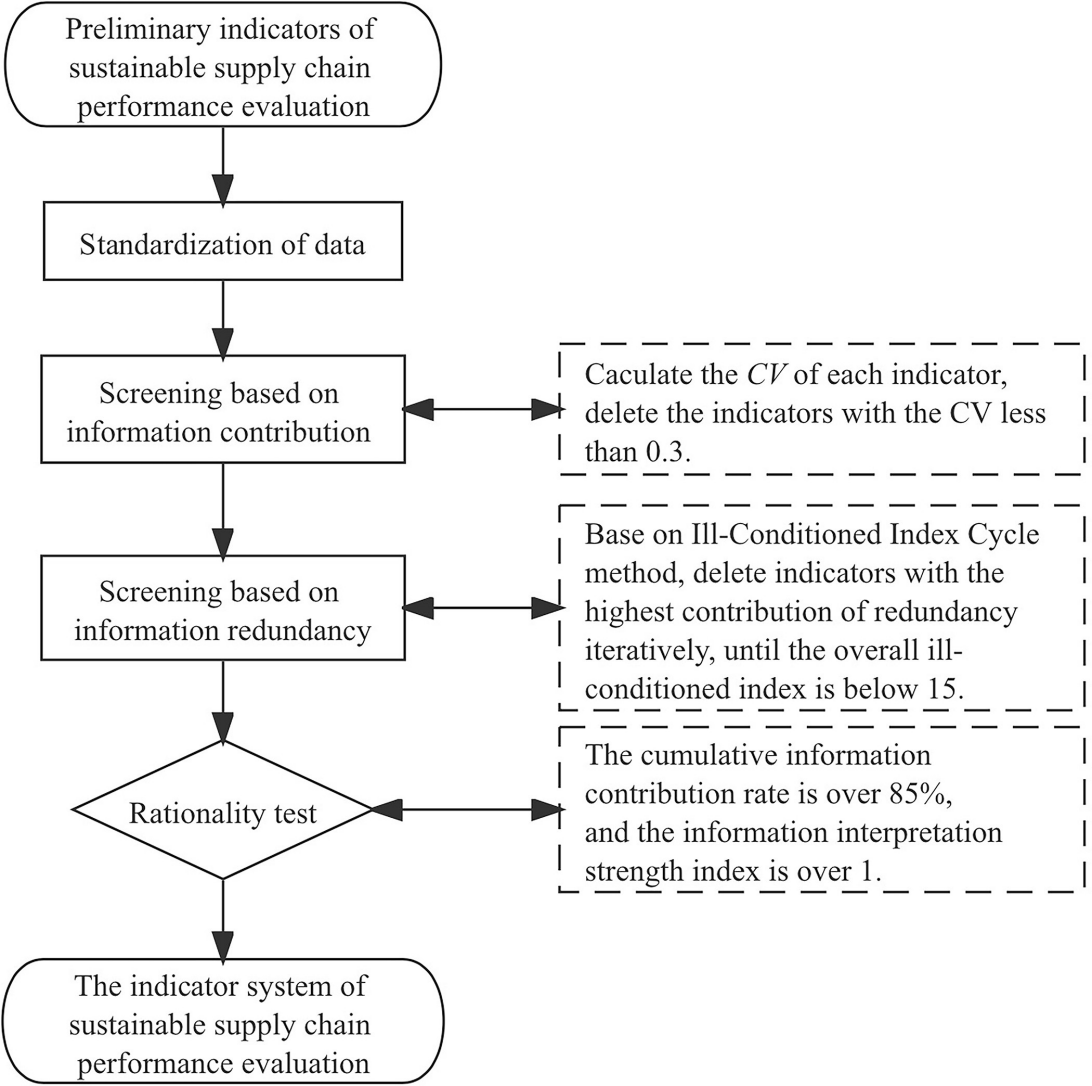
The quantitative screening procedure.

If the *I*S exceeds 1, the information interpretation intensity of the screened indicator system is stronger than that of the initial set. The rationality is verified when CR is more than 85%, and IS is more than 1. The overall procedure of screening is shown in **Fig 2.**

## Application of sustainable supply chain performance indicator screening

### Data source

Core listed companies at supply chain nodes have typically been chosen as samples in previous research because they have large-scale and network effects, and their publicly released consolidated financial statements contain large amounts of information about supply chains [40]. For this study, 100 listed companies in China were chosen as research samples; these companies represent the strong coverage of North, South, East, and West China, and cover typical industries, including agriculture, mining, manufacturing, electric power and gas, geological exploration, real estate, construction, pharmaceuticals, etc. Financial data were sourced from the CSMAR database from 2019–2021, and the social responsibility report rating results on relevant issues were sourced from the authoritative institution Run Ling Global (S1 Table). Small amounts of missing data were filled in using multiple imputation methods, and 300 research samples were generated.

### Data standardization

As determined in Step 1, the sample data were standardized by data categories (S2 Table), and condensed as depicted in Table 2.

**Table 2:** The standard values of the sample data.

Samples	*F1*	*F2*	*F3*	*F4*	.* *.* *.	*S4*	*S5*	*S6*	*S7*
Midea 2019	0.324	0.173	0.717	0.323	. . .	0.415	0.358	0.467	0.143
Midea 2020	0.310	0.154	0.711	0.320	. . .	0.468	0.420	0.404	0.226
Midea 2021	0.313	0.231	0.708	0.290	. . .	0.362	0.185	0.341	0.201
. . .	. . .	. . .	. . .	. . .	. . .	. . .	. . .	. . .	. . .
Aier 2019	0.628	0.251	0.703	0.329	. . .	0.437	0.691	0.670	0.685
Aier 2020	0.751	0.227	0.691	0.478	. . .	0.392	0.630	0.753	0.772
Aier 2021	0.588	0.256	0.702	0.317	. . .	0.196	0.593	0.581	0.706

### Indicator screening based on information contribution

**Fig 3:**
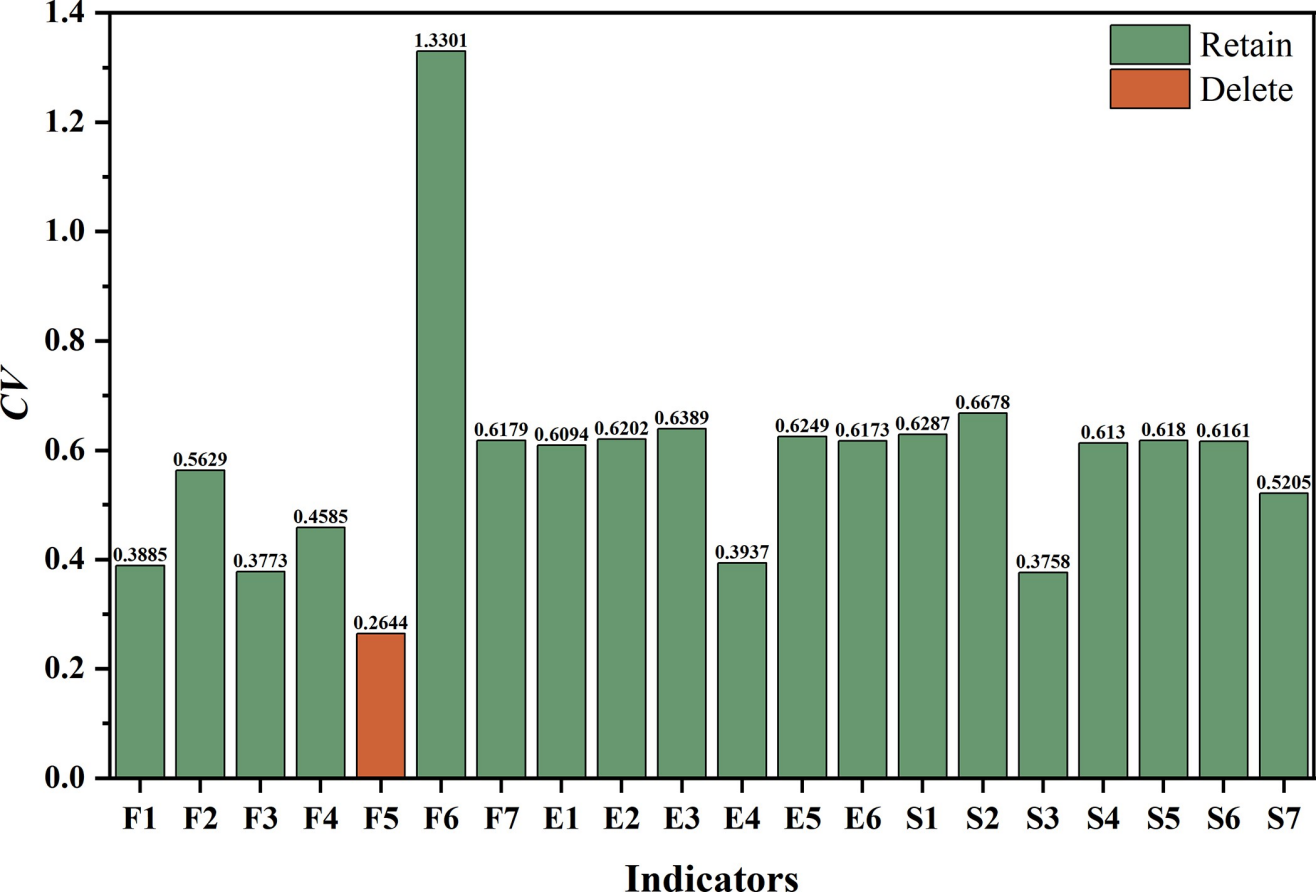
The results of indicator screening based on information contribution.

As stated in Step 2, the CV for each indicator was calculated based on standardized data (S3 Table). The findings are reported in **Fig 3.**

Based on the results, the *CV* of the indicator "Sustainable Growth Rate (*F5*)" from the economic dimension was 0.2644, which is less than 0.3, indicating that it should be deleted because its impact on the evaluation results is minimal. The remaining 19 indicators were kept.

The ill-conditioned index was calculated for the remaining indicator system according to Step 3, and the value of *CI*_*19*_ was 188.3796, reflecting serious information redundancy in the evaluation system. Redundancy screening was therefore necessary to streamline this indicator system.

### Indicator screening based on redundancy contribution

**Fig 4:**
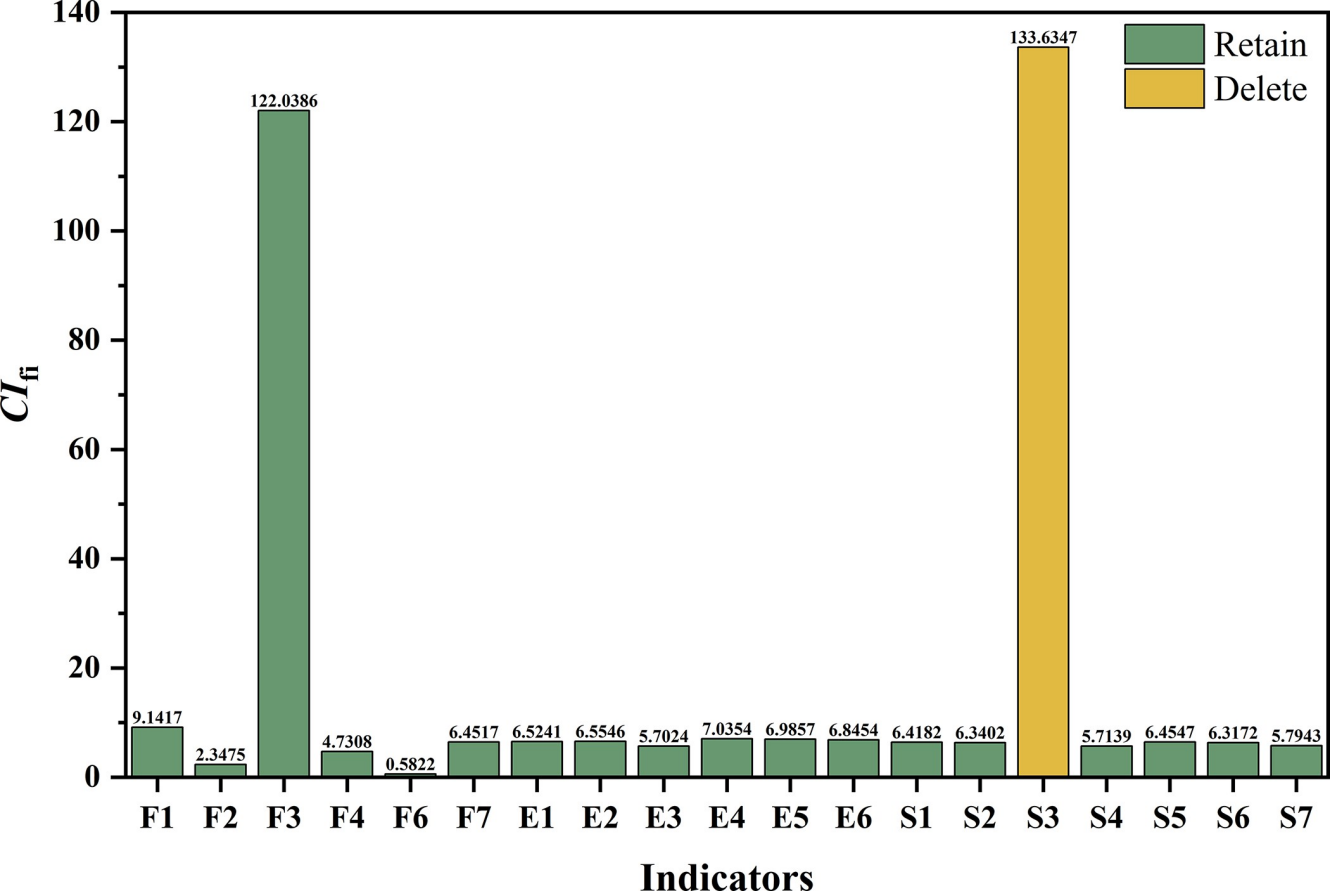
The results of the first round of redundancy screening.

The information redundancy contribution (*CI*_*fi*_) of the remaining 19 indicators was calculated according to Step 4. The findings are exhibited in **Fig 4.**

Based on the first round of redundancy screening results, the indicator "Emergency and Risk Management (*S3*)" of the social dimension was found to provide the highest redundancy contribution, with a *CI*_*s3*_ value of 133.6347. After removing it, the remaining indicator system had the lowest ill-conditioned index, with a *CI*_*18*_ value of 54.7449, reflecting the most inferior redundancy of the remaining 18 indicators. Therefore, the indicator "Emergency and Risk Management (*S3*)" was deleted in the first round of redundancy screening. At this point, the remaining indicator set still contained substantial information overlap, with the *CI*_*18*_ value being higher than the target threshold of 15, and a second round of redundancy screening was necessary.

**Fig 5:**
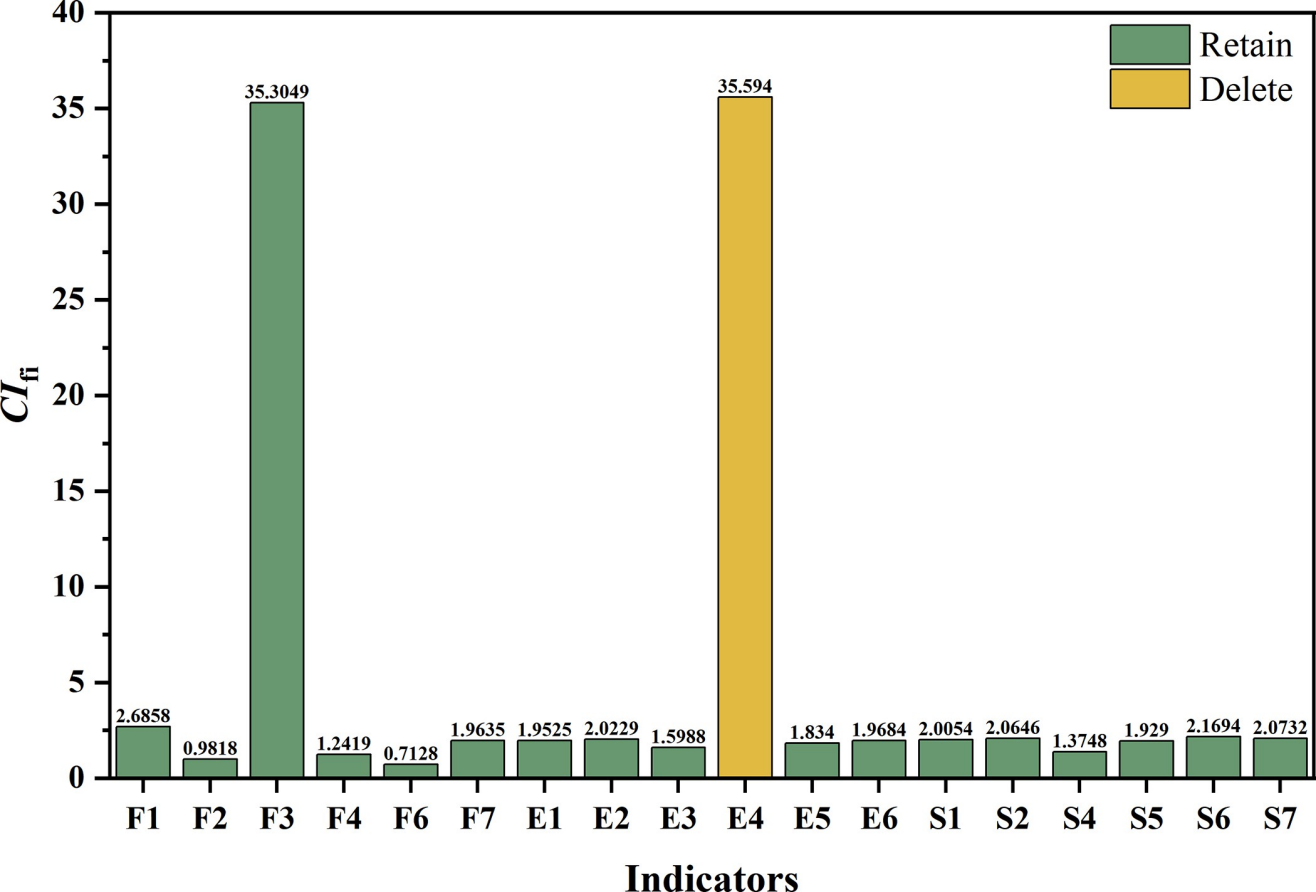
The results of the second round of redundancy screening.

The information redundancy contribution (*CI*_*fi*_) of the remaining 18 indicators was then calculated according to Step 4. The findings are reported in **Fig 5.**

Based on the results, the indicator "Discharge and Reuse (*E4*)" from the environmental dimension was found to provide the highest information redundancy contribution, with a *CI*_*e4*_ value of 35.5940. After removing it, the remaining indicator set had the lowest ill-conditioned index, with a *CI*_*17*_ value of 19.1510, indicating the minimal redundancy of the remaining 17 indicators. Therefore, the indicator "Discharge and Reuse (*E4*)" was deleted in the second round of redundancy screening. At this point, there remained moderate information overlap in the indicator system because the *CI*_*17*_ value was still above the target threshold of 15, so a third round of screening was needed.

**Fig 6:**
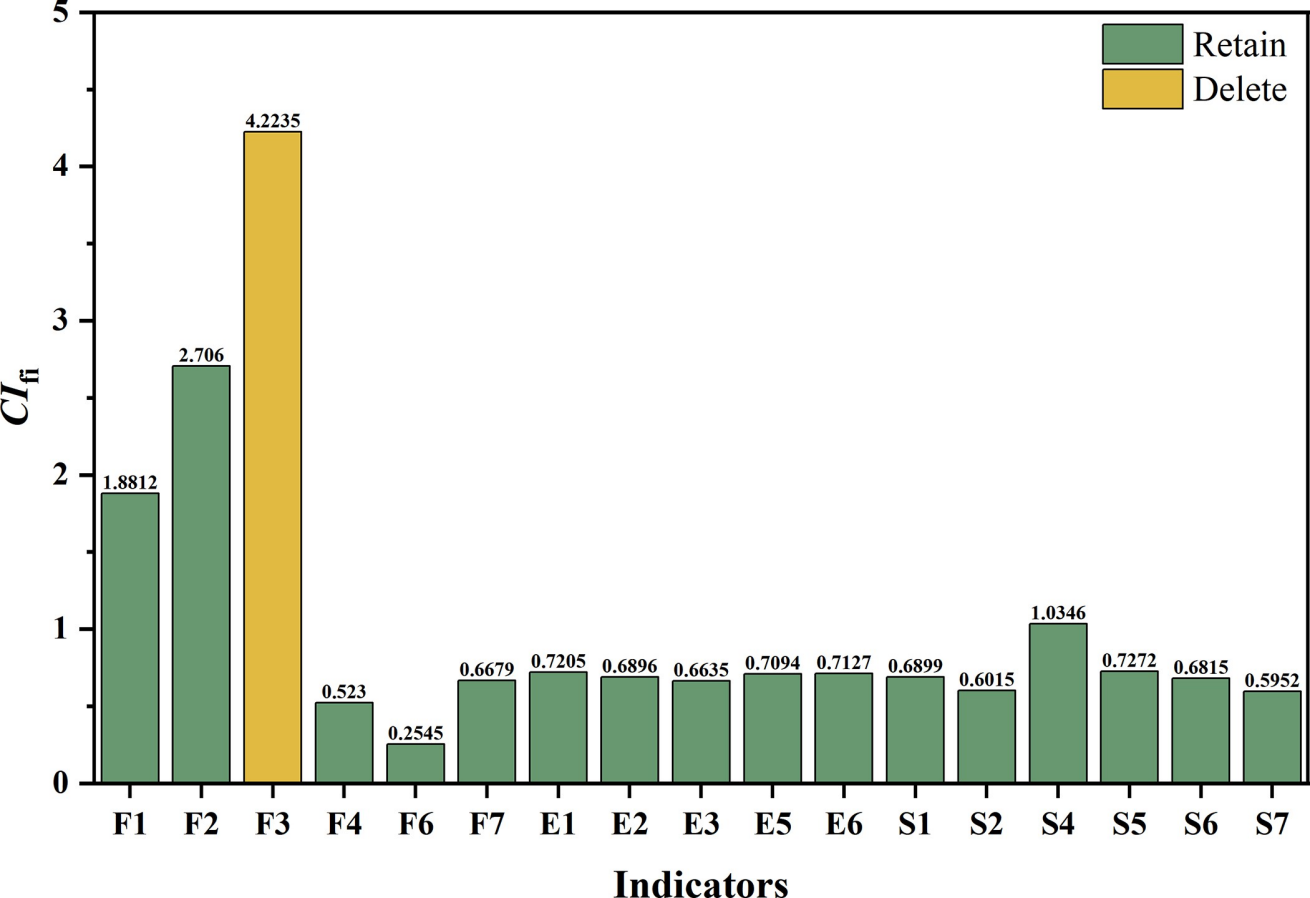
The results of the third round of redundancy screening.

Once again, the information redundancy contribution (*CI*_*fi*_) of the remaining 17 indicators was calculated according to Step 4. The findings are presented in **Fig 6.**

Based on the results, the indicator "Return on Equity Rate (*F3*)" of the economic dimension was found to provide the highest information redundancy contribution, with a *CI*_*f3*_ value of 4.2235. After removing it, the remaining indicator set had the lowest ill-conditioned index of 14.9275, reflecting minimal redundancy. Therefore, the "Return on Equity Rate (*F3*)" indicator was deleted. At this point, the redundancy screening was terminated, as the *CI*_*16*_ value had already fallen below the target threshold of 15 (S4 Table). This verifies that the redundancy of the indicator system was significantly reduced, and the information overlap of the remaining indicator system was slight.

### Rationality test

According to Step 5, the *CR* value of the screened indicator system was 87.77%, and the *IS* value was 1.2057. The outcome demonstrates that the information content of the final indicator system was higher than 85% of that of the initial selection after the screening, and its information interpretation intensity was 1.2057 times greater than that of the initial selection. Thus, beginning from significant information redundancy in the early proposed performance indicator system of sustainable supply chains, just slight redundancy was achieved in the system that was ultimately established. The indicators have a good capacity for information explanation and there is little information overlap, which fully supports the rationality of the findings of this study.

### Comparison with the conventional screening method

The coefficient of dispersion analysis is one of the widely used methods for indicator screening, since the *CV* is effective at identifying indicators with high information content. The results of the screening based on coefficient of dispersion analysis showed that the information contribution level of the indicator *F5* is too low, and the dispersion degree of the indicator *F6* is too high, so these two indicators need to be removed. The *CR* value of the indicator system derived from the proposed method and the coefficient of dispersion analysis was 87.77% and 86.19%, and the *IS* value was 1.2057 and 1.0821 (S5 Table).

The reduction ratio of redundancy contribution (*RR*) [32] is applied to illustrate the efficiency of reducing the overall information overlap for the indicator set. The equation of *RR* is as follows:

$$RR=\frac{{(CI}_{n}-{CI}_{n-k})/{CI}_{n}}{k}$$ (9)

Where *n* is the number of initially proposed indicators, and *k* is the number of indicators screened out.

The *RR* value of the proposed method and the coefficient of dispersion analysis was 30.04% and 0.31%, respectively. It demonstrates the effectiveness and superiority of the ill-conditioned index cycle method.

## Results and discussions

### Results analysis

Based on the connotation of a sustainable supply chain and TBL theory, the framework of the indicator system was constructed from the economic, environmental, and social dimensions. Concerning the standards issued by authoritative institutions and previous research, 53 indicators were proposed to form the evaluation system. According to the indicator selection principles, 33 indicators were removed because they lacked scientificity, representativeness, comparability, or obtainability. The *CV* was then used to identify the indicators with insufficient information contribution. Once an indicator was eliminated, the ill-conditioned index cycle method was employed to filter out the indicators contributing the most to information redundancy, and three indicators were deleted over three rounds. Then, a sustainable supply chain performance evaluation indicator system with 16 indicators was established. The ill-conditioned index of the indicator set dropped rapidly from 188.3796 to 14.9275, a sharp decline of 92.07%. The Pearson correlation coefficients between the indicators were all less than 0.5, indicating that the overall indicator set had very little redundancy and that the correlation between indicators was weak. The *CR* was 87.77% and the *IS* was 1.2057, thus supporting the rationality of the screening results and the scientific validity of the screening methodology. Moreover, comparing to the conventional coefficient of dispersion analysis, the *CR*, *IS*, and *RR* of the ill-conditioned index cycle method were all higher, indicating that the proposed method is more effective at identifying indicators with high information content and it shows the superiority at solving multicollinearity and reducing redundancy.

The final indicator system was sufficiently information-rich, had excellent information interpretation abilities, and exhibited high indicator differentiation. The established sustainable supply chain performance indicator system is shown in Table 3.

**Table 3:** The indicator system of sustainable supply chain performance evaluation.

Dimensions	Indicators	Type	Descriptions
**Economic**	Asset-Liability rate	N	Total liability / Total asset (Quantitative)
	Revenue growth rate	P	Revenue growth / Revenue of last period (Quantitative)
	Capital accumulation rate	P	Equity growth / Equity of the beginning balance (Quantitative)
	Inventory management	P	Operating costs / Average inventory (Quantitative)
	Logistics management	P	Rationality of product warehousing and transportation management. (Qualitative)
**Environmental**	Environmental management	P	Environmental supervision and assessment management. (Qualitative)
	Carbon intensity	N	Tons of carbon dioxide equivalent / Revenue (million RMB) (Quantitative)
	Energy consumption	N	Energy consumption of electricity, gas, and oil etc. (Qualitative)
	Product environmental impact	P	The impact of the products or services produced on the environment. (Qualitative)
	Renewable resource utilization	P	The utilization of renewable materials and energy in the production and operation process. (Qualitative)
**Social**	Compliance and business Ethics	P	Compliance with laws and regulations and anti-corruption measures. (Qualitative)
	Diversified collaborative governance	P	Diversified management models and collaborative governance of information technology. (Qualitative)
	Health and safety of Employee	P	Physical and mental health of employees and security guarantee. (Qualitative)
	Growth investment of employees	P	Investment in improving employee’s knowledge and skills. (Qualitative)
	Public welfare investment	P	Investment in poverty alleviation and public welfare, etc. (Qualitative)
	Social responsibility rankings	P	Scoring of social responsibility reports by the China Securities Investment Fund Industry Association (Quantitative)

### Limitations and future research

Nevertheless, this research was characterized by several limitations. Due to the potential impacts that accounting standards, carbon emissions disclosure, and social responsibility disclosure standards may have on the comparability of data, only Chinese-listed companies were chosen as the samples. Currently, Chinese listed companies are only partially required to disclose their social responsibility reports, which led to a small sample size for this study. Future research could explore this issue further by investigating more companies in different countries, such as Japan, Germany, and the United States because developing sustainable supply chains is a global issue, research findings may be more applicable when using diverse samples.

## Conclusions

Starting from the goal of the sustainable development of the supply chain, considering the implementing details of environmental and social factors, and regarding the most accepted international standards and high-quality studies from core collections, the indicators proposed in this study were characteristic and representative. Aiming at improving the contribution of evaluation indicators while efficiently reducing information overlap, the ill-conditioned index cycle method was used to systematically lower the degree of information overlap and minimize its detrimental effects on an accurate assessment. The methodology for quantitative screening successfully overcomes the shortcomings of subjective screening in earlier research. The outcome demonstrates that this screening method can strengthen the representativeness of the indicator system and lead to the better discrimination of the evaluation results. The final established sustainable supply chain performance indicator system can significantly reflect the current status of supply chain sustainability, and can also help guide enterprises to achieve their sustainable development goals of coordinated economic, environmental, and social benefits against the background of the "dual circulation" of the economy. Raw data of indicators

## Supporting information

S1 Table Raw data of indicators. (XLSX)

S2 Table Standardization of data. (XLSX)

S3 Table Screening based on information contribution. (XLSX)

S4 Table Screening based on information redundancy. (XLSX)

S5 Table Rationality test and comparison with conventional screening method. (XLSX)
